# A Novel Mechanism of the p53 Isoform Δ40p53α in Regulating Collagen III Expression in TGFβ1‐Induced LX‐2 Human Hepatic Stellate Cells

**DOI:** 10.1096/fj.202403146RR

**Published:** 2025-04-15

**Authors:** Sun‐Young Lee, Yunseong Jang, Hye‐Yeon Seok, Yong‐Hwan Moon

**Affiliations:** ^1^ Institute of Systems Biology Pusan National University Busan Korea; ^2^ Department of Integrated Biological Science Pusan National University Busan Korea; ^3^ Department of Molecular Biology Pusan National University Busan Korea

**Keywords:** Δ40p53 isoform, collagen III, ECM, LX‐2 human stellate cells, p53, Smad3, TGFβ1

## Abstract

Injured liver cells undergoing chronic wound healing produce excessive amounts of extracellular matrix (ECM) components, such as collagen and fibronectin, leading to fibrosis. This process is largely mediated by transforming growth factor‐β (TGFβ) signaling, which intersects with the tumor suppressor p53 pathway. However, the roles of specific p53 isoforms in this interaction remain unclear. In this study, we report the involvement of the Δ40p53α isoform, an N‐terminal truncated variant of p53, in regulating ECM gene expression in TGFβ1‐activated LX‐2 human hepatic stellate cells. RT‐PCR analysis of cirrhotic liver tissues revealed clinically relevant increases in Δ40p53 expression. Knockdown of Δ40p53 using antisense oligonucleotides in LX‐2 cells attenuated TGFβ1‐induced activation and significantly reduced collagen production and deposition, particularly fibrillar collagen III. Conversely, overexpression of Δ40p53α upregulated collagen III expression in concert with full‐length p53 (FLp53). Co‐immunoprecipitation analysis demonstrated that Δ40p53α forms a complex with FLp53, which associates with phosphorylated Smad3 following TGFβ1 stimulation. These findings suggest that Δ40p53 enhances collagen III expression by interacting with FLp53 and Smads, highlighting its role in profibrogenic ECM expression.

## Graphical abstract

Δ40p53α, an N‐terminal truncated isoform of p53, regulates extracellular matrix gene expression in TGFβ1‐activated LX‐2 hepatic stellate cells. Upon liver injury, TGFβ1 signaling induces Δ40p53α, promoting its complex formation with full‐length p53 (FLp53). This complex associates with phosphorylated Smad3 in the nucleus, enhancing COL3A1 transcription and increasing collagen III deposition. These events contribute to excessive ECM accumulation, highlighting a key regulatory mechanism in the fibrogenic process.

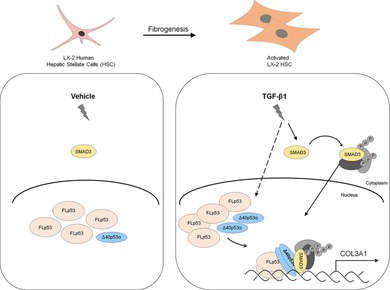

**Abbreviations**
2′‐OMe: 2′‐O‐methylation
ASO: antisense oligonucleotide
Co‐IP: co‐immunoprecipitation
CTGF: connective tissue growth factor
DAPI: 4′,6‐diamidino‐2‐phenylindole
DMEM: Dulbecco's Modified Eagle Medium
ECM: extracellular matrix
GAPDH: glyceraldehyde‐3‐phosphate dehydrogenase
HSCs: hepatic stellate cells
IRES: internal ribosome entry site
MMPs: matrix metalloproteinases
PAI‐1: plasminogen activator inhibitor‐1
qRT‐PCR: quantitative RT‐PCR
TBS: Tris‐buffered saline
TGFβ: transforming growth factor‐β
TIMPs: tissue inhibitors of metalloproteinases
α‐SMA: α‐smooth muscle actin

## 1 Introduction

The extracellular matrix (ECM) is essential for maintaining tissue‐specific functions and structural integrity. The homeostasis of ECM relies on a balance between its production, deposition, and degradation. Disruption of this balance is a key driver of pathological conditions such as liver fibrosis. During fibrogenesis, damaged liver cells form scar tissue and deposit excessive amounts of ECM components, including collagen, fibronectin, and other matrix components [1]. Identifying the key regulators of fibrogenic processes and elucidating the molecular mechanisms underlying profibrogenic ECM expression are crucial for the development of effective therapeutic interventions.

Hepatic stellate cells (HSCs) are the primary source of ECM in liver fibrosis, constituting 5%–10% of the total liver population [2, 3]. In healthy livers, HSCs are quiescent and regulate ECM turnover [1]. However, in response to chronic injury, multiple cell types, including hepatocytes and macrophages, release inflammatory cytokines. These cytokines activate HSCs, triggering their transdifferentiation into myofibroblast‐like phenotypes characterized by proliferation, contractility, and migratory properties [4]. Activated HSCs are primarily responsible for liver fibrosis by overproducing and depositing ECM, particularly fibrillar collagens such as collagen types I and III [5]. Increased levels of cross‐linked fibrillar collagen contribute to tissue fibrosis.

Transforming growth factor‐β (TGF‐β) is the most potent profibrogenic cytokine, mediating diverse cellular responses, including epithelial‐mesenchymal transition, ECM production, cell proliferation, and apoptosis [6]. TGF‐β signaling pathways are involved in the profibrogenic processes in the liver through both canonical and non‐canonical pathways. In the canonical pathway, ligand binding to the TGF‐β receptor induces phosphorylation of Smad2/3, which translocates to the nucleus to regulate fibrogenic gene transcription. The non‐canonical pathways involve mitogen‐activated protein kinase, Rho‐like GTPase, and phosphatidylinositol‐3‐kinase/AKT signaling [7, 8]. In addition, the TGF‐β signaling pathway intersects with the tumor suppressor protein p53. For example, in *Xenopus* embryonic development, p53 regulates the activation of TGF‐β target genes by cooperating with Smads [9]. TGF‐β‐induced plasminogen activator inhibitor‐1 (PAI‐1) gene expression is regulated by the p53/Smad complex [10, 11]. These studies highlight the potential role of p53 in modulating TGF‐β‐mediated ECM gene expression.

The tumor suppressor protein p53 is well known for its pivotal role in regulating apoptosis, senescence, and cell‐cycle arrest in response to cellular stress [12, 13]. Despite extensive research, the role of p53 and its isoforms in profibrogenic signaling remains underexplored. Evidence highlights p53 as a key mediator of various genes associated with both profibrogenic and antifibrogenic processes, with its roles varying by context and tissue type [14, 15]. These genes include hepatocyte growth factor, PAI‐1, collagen I, fibronectin, collagen IV, collagens IIa1 and VIa1, and metalloproteinases [16, 17, 18, 19, 20, 21, 22, 23]. Furthermore, elevated p53 expression induces the production of connective tissue growth factors (CTGFs) in hepatocytes, which drive fibrotic processes [24]. These findings underscore the complex, context‐dependent roles of p53.

**FIGURE 1:**
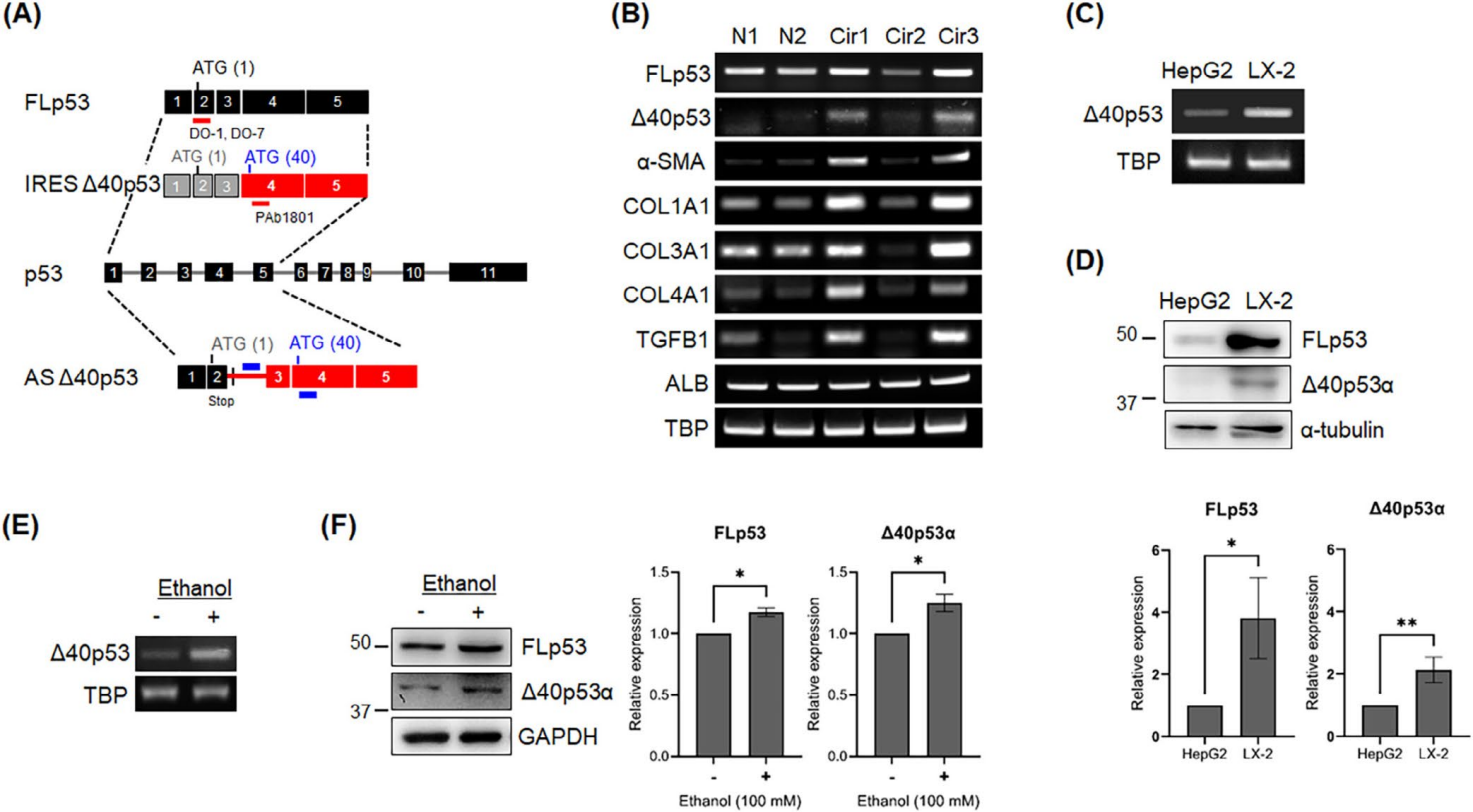
Δ40p53 expression is associated with liver fibrosis. (A) Scheme representation of TP53 gene organization and mechanisms of Δ40p53 production. The first five exons, corresponding to the N‐terminal region of the full‐length (FLp53) and the Δ40p53 isoform transcripts, are highlighted. Exons are shown as black boxes, with introns represented by lines between them. Red boxes indicate exons involved in producing the Δ40p53 isoform. Alternative splicing (AS) Δ40p53 results in retention of intron 2. An alternative mechanism for Δ40p53 production via internal ribosomal entry site (IRES)‐mediated translation is also depicted, with gray boxes indicating exons excluded from the IRES‐mediated translation. Two ATG start codons are indicated, corresponding to the translation initiation sites for FL p53 and Δ40p53, respectively. The red bars represent regions recognized by the p53 antibodies used in this study, and the blue bars indicate primer target regions for detecting the Δ40p53 transcript. (B) Representative RT‐PCR data showing gene expression pattern of Δ40p53 in normal (N) and cirrhotic (Cir) patients' samples. The albumin (ALB) gene was used to confirm the identification of liver tissue, and TATA‐binding protein (TBP) gene was used as an internal control. (C) Representative semi‐quantitative RT‐PCR showing mRNA expression of Δ40p53 in HepG2 and LX‐2 cells. TBP was used as an internal control. (D) Western blot analysis and quantification of endogenous Δ40p53 protein levels in HepG2 and LX‐2 cells. α‐tubulin served as a loading control. The blots are representative of three independent experiments. (E) Representative semi‐quantitative RT‐PCR showing mRNA expression of Δ40p53 in LX‐2 cells treated with 100 mM ethanol, with fresh ethanol added daily for seven consecutive days. TBP was used as an internal control. (F) Western blot analysis and quantification of endogenous Δ40p53 protein levels in LX‐2 cells treated with 100 mM ethanol, with daily ethanol replenishment for 7 days. α‐tubulin served as a loading control. Blots represent three independent experiments. All quantitative data are presented as mean ± SD. *p < 0.05; **p < 0.001 [two‐tailed unpaired *t*‐test (D, F)].

To date, 12 p53 isoforms have been identified, and numerous studies have accumulated to elucidate the functions of these isoforms [25]. Among them, the Δ40p53 isoform is a naturally truncated isoform that lacks 39 amino acids from the N‐terminal region. The Δ40p53 isoform retains the second transactivation domain and lacks the Mdm2‐binding site [26, 27]. Oligomerization with full‐length p53 (FLp53) stabilizes FLp53 against Mdm2‐mediated degradation [28], and FLp53‐Δ40p53 complexes respond differently depending on the cell stress context [29]. The Δ40p53 protein is produced through alternative splicing that retains intron 2 [30] or through an internal ribosome entry site (IRES)‐mediated alternative translational mechanism [31, 32, 33] that uses the initiation codon AUG 40 in exon 4 instead of AUG 1 in the fully spliced p53 mRNA (Figure 1A). In addition, Δ40p53 can generate three different isoforms through classical (Δ40p53α, also known as p47 and ΔNp53) and alternative (Δ40p53β and Δ40p53γ) splicing of the C‐terminus of the p53 gene [34]. Studies show that Δ40p53 not only modulates FLp53 activity by forming complexes but also possesses distinct functional properties [26, 29, 33, 35, 36, 37, 38], making it a critical player in the cellular stress response and potentially in profibrogenic processes.

This study focused on the Δ40p53α isoform and its role in regulating profibrogenic ECM expression, specifically its effect on collagen III expression in TGFβ1‐activated LX‐2 human stellate cells. LX‐2 cells were chosen because they are a widely accepted in vitro model for studying HSC activation and fibrogenic pathways. TGFβ1, the major profibrogenic cytokine, was used as an experimental activator due to its relevance to profibrogenic conditions in vivo.

We report that Δ40p53α regulates collagen III expression under profibrogenic conditions by interacting with FLp53 and Smad3. To our knowledge, this is the first study to demonstrate a specific role for Δ40p53α in regulating ECM remodeling under profibrogenic conditions. These findings provide novel insights into the molecular mechanisms underlying TGFβ1‐induced ECM expression and open up new avenues for potential therapeutic strategies for addressing chronic liver diseases.

## 2 Materials and Methods

### 2.1 Cell Lines

LX‐2 human HSCs were purchased from Merck Millipore (Cat# SCC064, RRID:CVCL_5792) and cultured in Dulbecco's Modified Eagle Medium (DMEM), supplemented with 2% FBS (GIBCO, Waltham, MA, USA), 2 mM Glutamine (GIBCO), and antibiotics (50 U/mL penicillin and 50 μg/mL streptomycin). Human hepatoblastoma HepG2 cells were obtained from the Korean Cell Line Bank (Cat# 88065, RRID:CVCL_0027) and grown in DMEM supplemented with 10% FBS and antibiotics. Cells were periodically tested for mycoplasma contamination using the BioMycoX mycoplasma PCR detection kit (CellSafe, Yongin, Korea).

### 2.2 Antibodies and Reagents

The following antibodies were used: anti‐p53 (Santa Cruz Biotechnology DO‐1 [Cat# sc‐126, RRID:AB_628082], DO‐7 [Cat# sc‐47 698, RRID:AB_628083], PAB1801 [Cat# sc‐98, RRID:AB_628085], and Cell Signaling Technology 7F5 [Cat# 2527, RRID:AB_10695803]), anti‐collagen I (Proteintech Cat# 14695‐1‐AP, RRID:AB_2082037), anti‐collagen III (Proteintech Cat# 22734‐1‐AP, RRID:AB_2879158), anti‐collagen IV (Abcam Cat# ab6586, RRID:AB_305584), anti‐α‐SMA (Sigma‐Aldrich Cat# A5228, RRID:AB_262054), anti‐GAPDH (Proteintech Cat# 60004‐1‐Ig, RRID:AB_2107436), anti‐α‐tubulin (Sigma‐Aldrich Cat# CP06, RRID:AB_2617116), anti‐Myc Tag (4A6, agarose conjugate, Millipore Cat# 16‐219, RRID:AB_390197), anti‐Myc Tag (Thermo Fisher Scientific Cat# PA1‐981, RRID:AB_325961), anti‐Smad3 (Cell Signaling Technology Cat# 9523, RRID:AB_2193182), and anti‐p‐Smad3 (Cell Signaling Technology Cat# 9520, RRID:AB_2193207). TGF‐β1 was purchased from PEPROTECH (Cranbury, NJ, USA). RNAiMAX (Thermo Fisher Scientific, Waltham, MA, USA) and FuGENE 6 (Promega, Madison, WI, USA) were used for transfection.

### 2.3 Total RNA of Liver Tissues

Total RNA samples from normal human and cirrhotic liver tissues, diagnosed with pathological verification, were purchased from OriGene Technologies (Rockville, MD, USA).

### 2.4 Antisense Oligonucleotides and Transfection

The chemically synthesized gapmer antisense oligonucleotides (ASOs) consisted of 20 nucleotides, with 5′ and 3′ flanking segments containing 2′‐O‐methoxyethyl bases and a central DNA segment spanning 10 bases, all modified with phosphorothioate (Integrated DNA Technologies Inc., Newark, NJ, USA). The steric blocking ASOs (control and Δ40p53 2′‐OMe ASO) were synthesized as described by Swiatkowska et al. [39]. The target sequences are listed in Table 1. LX‐2 cells were plated 24 h before transfection at a density of 2 × 10^4^ cells/cm^2^. The cells were then transfected with ASOs using RNAiMAX (Thermo Fisher Scientific), following the manufacturer's instructions. FAM‐labeled oligonucleotides (FAM‐luciferase) were transfected and visualized using fluorescence microscopy to assess the transfection efficiency.

**TABLE 1:** Antisense oligonucleotides (ASO).

ASO name	Sequences (5′ → 3′)
Control gapmer	ACCAGGGCGTATCTCTCCAT
Δ40 gapmer	CAGTCCCATGGAATTTTCGC
Control‐2′‐OMe	ACCAGGGCGUAUCUCUCCAUA
Δ40‐2′‐OMe	AACGUUGUUUUCAGGAAGUAG
FAM‐luciferase	ACCAGGGCGTATCTCTCCATA

### 2.5 Plasmids

The Δ40p53α and FLp53 overexpression plasmids, pSV40‐Δ40p53α‐Myc, were constructed using the pGL3 promoter (a gift from Debrya Groskreutz, Addgene plasmid #212939; http://n2t.net/addgene:212939; RRID: Addgene_212 939) and pCMV‐FLp53 (p53α)‐Myc was constructed using pCDNA 3.1(+) (Invitrogen, Waltham, MA, USA) vectors, respectively.

The primers used for pSV40‐Δ40p53α‐Myc were 5′‐AAGCTTATGGATGATTTGATGCTG‐3′ and 5′‐TCTGAGTCACAGATCCTCTTCTGAGATGAGTTTTTGTTCGTCTGAGTCAGGCCCTTCTGT‐3′. The primers used for pCMV‐FLp53‐Myc were 5′‐AGATCTATGGAGGAGCCGCAGTCAGA‐3′ and 5′‐TCTGAGTCACAGATCCTCTTCTGAGATGAGTTTTTGTTCGTCTGAGTCAGGCCCTTCTGT‐3′. The human COL3A1 promoter region from −1,100 to +35 was amplified from the genomic DNA of LX‐2 cells using the primers 5′‐GGTACCGATTATAGAGATAGATGA‐3′ and 5′‐AGATCTCCCTTCAGCACCGGGCCC‐3′, and cloned into pGL3‐Basic (Promega) to generate the COL3A1‐luciferase reporter vector. pCMV5B‐Flag‐Smad3 was a gift from Jeff Wrana (Addgene plasmid #11742; http://n2t.net/addgene:11742; RRID: Addgene_11742) [40].

### 2.6 Quantitative and Semi‐Quantitative RT‐PCR

Quantitative RT‐PCR (qRT‐PCR) was performed on a QuantStudio real‐time PCR system using 2× POWER SYBR Green PCR Master mix (Applied Biosystems, Cat# 4367659, Waltham, MA, USA). Total RNA was extracted using QIAshredder (Qiagen, Tegelen, Netherlands) and the RNAqueous Total RNA Isolation Kit (Thermo Fisher Scientific), then transcribed into cDNA using oligo (dT) primers and M‐MLV reverse transcriptase (Promega). The primer sequences used for RT‐PCR are listed in Table 2.

**TABLE 2:** The list of primer sequences used for RT‐PCR.

Gene	Forward primers (5′ → 3′)	Reverse primers (5′ → 3′)
Δ40p53	ACCAGGGCGTATCTCTCCAT	TCTGAAAGACAAGAGCAGAA
Fully spliced p53	ATGGAGGAGCCGCAGTCAGAT	GCAGCCTCTGGCATTCTGGGA
COL1A1	AGCCAGCAGATCGAGAACAT	TCTTGTCCTTGGGGTTCTTG
COL3A1	AGGGGAGCTGGCTACTTCTC	TAGGAGCAGTTGGAGGCTGT
COL4A1	GAAGGGTGATCCAGGTGAGA	CACCCTTGTCACCTTTTGGT
ACTA2	AATGGCTCTGGGCTCTGTAA	TCTTTTCCATGTCCCAGTTG
TGFB1	CACGTGGAGCTGTACCAGAA	GAACCCGTTGATGTCCACTT
ALB	CTCAAGTGTGCCAGTCTCCA	TGGGATTTTTCCAACAGAGG
MMP‐1	GATGAAGCAGCCCAGATGTG	GCTTGACCCTCAGAGACCTT
MMP‐2	TCTCCTGACATTGACCTTGGC	CAAGGTGCTGGCTGAGTAGAT
MMP‐9	TTGACAGCGACAAGAAGTGG	GCCATTCACGTCGTCCTTAT
TBP	TATAATCCCAAGCGGTTTGC	GCAGGAAAACCCAACTTCTG
GAPDH	ACCCACTCCTCCACCTTT	CTCTTGTGCTCTTGCTGGG

### 2.7 Co‐Immunoprecipitation and Western Blotting

Cells were lysed in either NP‐40 buffer (50 mM Tris pH 7.4, 250 mM NaCl, 5 mM EDTA, 1 mM NaF, 1 mM Na_3_VO_4_, 1% Nonidet‐40, and protease and phosphatase inhibitor cocktails) or modified RIPA buffer (50 mM Tris pH 7.6, 150 mM NaCl, 1 mM EDTA, 1 mM EGTA, 1% Triton‐X 100, and protease and phosphatase inhibitor cocktails). The lysates were centrifuged at 10 000 *g* at 4°C for 20 min. Two hundred micrograms of cell lysates were incubated with 2 μg of antibody for 4 h at 4°C for co‐immunoprecipitation. The samples were then incubated with Protein A/G magnetic beads (Thermo Fisher Scientific, Cat# 88802) for 1 h at room temperature, followed by three washes with 1 mL of Tris‐buffered saline (TBS) containing 0.05% Tween‐20 detergent. The bead‐protein complexes were incubated for 5 min either at 70°C or 98°C with Laemmli sample buffer. Protein samples were loaded onto a pre‐cast 10% or 4%–12% tris‐glycine polyacrylamide gel using the NOVEX gel system (Thermo Fisher Scientific). The resolved proteins were transferred to PVDF membranes (Millipore), followed by blocking in TBS and 0.1% Tween‐20 with 5% skim milk. Membranes were incubated in TBS, 0.1% Tween‐20, and 5% skim milk containing primary antibodies. The primary antibodies were detected using an HRP conjugated anti‐mouse or rabbit IgG antibody (Thermo Fisher Scientific Cat# 31430, RRID:AB_228307 and Cat# 31460, RRID:AB_228341) and an ECL detection kit (Cytiva, Marlborough, MA, USA). Chemiluminescent signals were captured using a Fusion Solo S imaging system (Vilber Lourmat, Marne La Vallee, France).

### 2.8 Immunofluorescence Staining

Cells were fixed in Methanol: Acetone (50:50) solution for 10 min at −20°C and blocked with 3% bovine serum albumin (BSA). The samples were incubated with the primary antibodies diluted in IF buffer (137 mM NaCl, 10 mM Na_2_HPO_4_, 1.8 mM KH_2_PO_4_, 0.1% BSA, 0.2% Triton X‐100, and 0.05% Tween‐20) overnight at 4°C in a humidified chamber. After intensive washing in IF buffer, the samples were incubated with Alexa Fluor dye‐coupled secondary antibodies (Invitrogen) for 1 h at room temperature. Nuclei were stained with 1 μg/mL 4′,6‐diamidino‐2‐phenylindole (DAPI), and the samples were rinsed with PBS and mounted with Fluoremount‐G (Electron Microscopy Sciences, Hatfield, PA, USA).

### 2.9 Luciferase Reporter Assay

Cells were co‐transfected with the pGL3‐COL3A1‐firefly luciferase reporter plasmid and SV40‐*Renilla* luciferase (pRL‐SV40; Promega) in combination with FLp53 or Δ40p53α isoform in a 12‐well plate. The transfection was performed in duplicate with 1 μg of the pGL3‐CPL3A1‐firefly luciferase reporter vector, 0.05 μg of SV40‐Renilla luciferase, and either 0.5 μg of CMV‐FLp53, SV40‐Δ40p53α, or a combination of both. The pUC19 plasmid (New England Biolabs Cat# N3041S, Ipswich, MA, USA) was used to adjust the DNA concentration in each transfection mixture. Twenty‐four hours after transfection, the cells were lysed, and a luciferase assay was performed using the Dual‐Luciferase Assay System (Promega) according to the manufacturer's instructions. Firefly and *Renilla* luciferases were measured using GloMax (Promega), and firefly reporter activity was normalized to *Renilla* luciferase activity.

### 2.10 Transfection

To avoid unintended long‐term stress responses, the cells were transiently transfected using FuGENE 6 (Promega), following the manufacturer's instructions.

### 2.11 Statistical Analyses

All values are presented as the mean ± SD. GraphPad Prism (version 10.3 RRID:SCR_002798) was used for the statistical analyses. Statistical significance was determined using an unpaired two‐tailed Student's *t*‐test, one‐way analysis of variance (ANOVA), or two‐way ANOVA with Tukey's or Dunnett's post hoc tests, as described in the figure legends. *p* < 0.05 were considered statistically significant.

## 3 Results

### 3.1 Elevated Δ40p53 Expression Is Associated With Liver Fibrosis

To evaluate the clinical significance of the Δ40p53 isoforms in fibrotic liver tissues, we surveyed Δ40p53 RNA expression in liver samples from patients with pathologically normal or cirrhotic livers. Since the three Δ40p53 isoforms (Δ40p53α, β, and γ) are produced from Δ40p53 transcripts that retain intron 2 of the TP53 gene, our primer set can detect these isoforms. Therefore, we collectively refer to the three Δ40p53 isoforms as Δ40p53 when analyzing RNA transcript data. We observed elevated levels of Δ40p53 transcripts in two out of three cirrhotic liver tissues analyzed, alongside increased expression of established molecular markers of cirrhosis, including α‐smooth muscle actin (ACTA2), collagen type I (COL1A1), III (COL3A1), and IV (COL4A1) genes. Normal liver samples exhibited only marginal expression of Δ40p53 compared with the cirrhotic samples (Figure 1B). The liver tissues that showed the increased Δ40p53 transcripts were pathologically determined to be chronic inflammatory infiltrates (Table S1), indicating that Δ40p53 is positively correlated with the inflammatory stress response. Further examination of Δ40p53 expression at both RNA and protein levels revealed higher levels in the immortalized LX‐2 human HSC line than in hepatoblastoma HepG2 cells, which are known to exhibit most cellular features of normal human hepatocytes (Figure 1C,D). The Δ40p53 isoform expressed in LX‐2 cells was identified as Δ40p53α, based on its molecular weight (47 kDa). This suggests that Δ40p53α may play more significant roles in HSCs, which are crucial contributors to the profibrogenic process, compared to hepatocytes. Additionally, we observed an increase in Δ40p53α expression in LX‐2 cells treated with 100 mM ethanol for seven consecutive days (Figure 1E,F), indicating that Δ40p53α expression is upregulated in response to liver damage.

**FIGURE 2:**
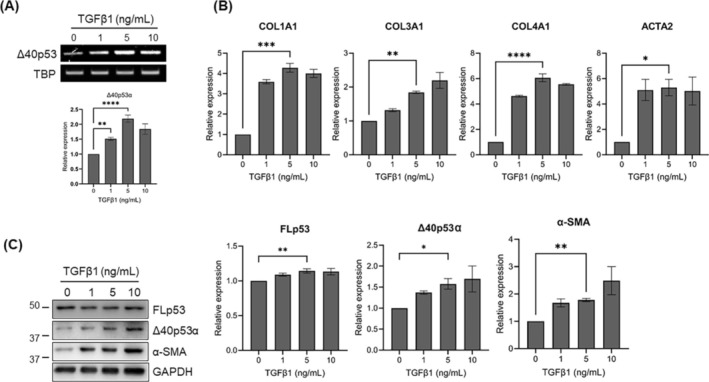
The expression of Δ40p53 increases in TGFβ1‐stimulated LX‐2 HSC cells. (A) Representative semi‐quantitative RT‐PCR showing mRNA expression of Δ40p53 in HSC cells stimulated with increasing concentrations of TGFβ1 for 24 h. TBP was used as an internal control. (B) qRT‐PCR analysis of fibrogenic gene expression, including ECM molecules (COL1A1, COL3A1, and COL4A1) and α‐smooth muscle Actin (ACTA2), in HSC cells exposed to increasing concentrations of TGFβ1 for 24 h. (C) Western blot analysis and quantification of endogenous FLp53, Δ40p53, and α‐SMA in response to TGFβ1 stimulation. Blots represent three independent experiments. GAPDH served as a loading control. The full‐blot images of FLp53 and Δ40p53 are shown in Figure S1A. All quantitative data are presented as mean ± SD. *p < 0.05; **p < 0.001; ***p < 0.0001; ****p < 0.00001 [one‐way ANOVA with Tukey's multiple comparison tests (B, C)].

We next examined if stimulating LX‐2 cells with recombinant human TGFβ1 would alter Δ40p53 expression. Semi‐quantitative RT‐PCR revealed that Δ40p53 mRNA expression is increased by 1.5‐ to 2‐fold upon TGFβ1 stimulation (Figure 2A). LX‐2 activation was confirmed by increased expression of COL1A1 (3.5‐ to 4‐fold), COL3A1 (2‐fold), COL4A1 (6‐fold), and ACTA2 (5‐fold), as measured by quantitative RT‐PCR (Figure 2B). Additionally, western blot analysis demonstrated that the Δ40p53α protein was upregulated moderately yet consistently in a dose‐dependent manner, while the canonical FLp53 was marginally increased upon TGFβ1 stimulation (Figure 2C, and Figure S1A). Taken together, these data suggest that Δ40p53α expression is upregulated by liver damage and in response to TGFβ1, potentially participating in TGFβ1‐mediated signaling pathways in human HSCs.

### 3.2 Downregulation of Δ40p53, Which Retains Intron 2, Affects the Expression of COL3A1 and ACTA2, and Influences ECM Remodeling

**FIGURE 3:**
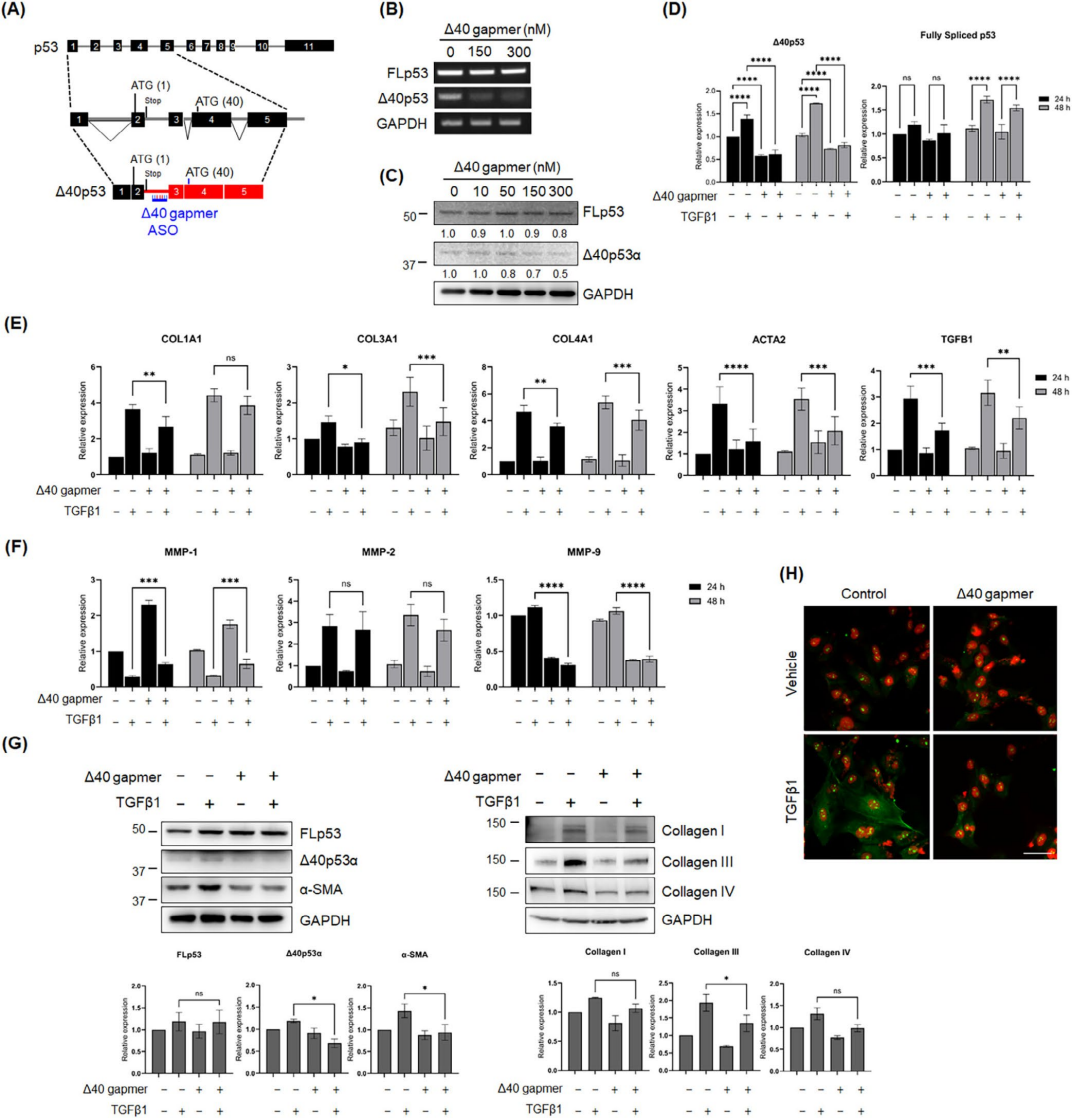
ASOs targeting Δ40p53 inhibit LX‐2 cell activation and reduce the expression of profibrogenic genes. (A) Schematic of the Δ40p53 isoform production through alternative splicing. The ASO target region (Δ40 Gapmer) is indicated. (B) Representative semi‐quantitative RT‐PCR showing Δ40p53 expression in LX‐2 cells treated with varying concentrations of Δ40 gapmer. GAPDH was used as an internal control. (C) Western blot analysis of Δ40p53 levels in LX‐2 cells treated with the indicated concentrations of Δ40 gapmer. GAPDH served as a loading control. (D–F) qRT‐PCR analysis of mRNA expression levels in control gapmer‐ and Δ40 gapmer‐treated LX‐2 cells stimulated with either vehicle or 5 ng/mL TGFβ1 for 24 and 48 h (*N* = 3). (D) Expression of Δ40p53 and fully spliced p53. (E) Expression of profibrogenic genes. (F) Expression of MMP genes. GAPDH was used as an internal control. (G) Western blot analysis and quantification of FLp53, Δ40p53, α‐SMA, and collagen I, III, and IV in control gapmer‐ and Δ40 gapmer‐treated LX‐2 cells stimulated with either vehicle or 5 ng/mL TGFβ1 for 24 h. Blots represent three independent experiments. GAPDH served as a loading control. (H) Representative immunofluorescence images of α‐SMA (green) in control gapmer‐ and Δ40 gapmer‐treated LX‐2 cells stimulated with either vehicle or 5 ng/mL TGFβ1 for 24 h. Nuclei are counterstained with DAPI and pseudocolored red for enhanced contrast. Scale bar, 25 μm. All quantitative data are presented as mean ± SD. *p < 0.05; **p < 0.001; ***p < 0.0001; ****p < 0.00001; ns, not significant [two‐way ANOVA with a Tukey's multiple comparison (D–F); one‐way ANOVA with a Dunnett's multiple comparison test (G)].

We used a loss‐of‐function approach to determine whether the fibrogenic process was disrupted when Δ40p53 expression was reduced. To this end, we employed gapmer ASOs to induce RNase H‐mediated degradation of Δ40p53 transcripts. Δ40p53 retains intron 2 and creates in‐frame stop codons, which, in turn, prevent the translation of FLp53 and lead to the generation of Δ40p53 isoforms using an AUG 40 initiation codon in exon 4 [30]. The specific gapmer ASO (referred to as the Δ40 gapmer hereafter) was designed to target Δ40p53 transcripts retaining intron 2 (Figure 3A). We first measured the inhibitory effect of the Δ40 gapmer on Δ40p53α expression in LX‐2 cells at various concentrations and observed efficient knockdown at a dose of 150 nM (Figure 3B,C). Notably, we found that the protein level of Δ40p53α was less affected, despite significant downregulation of its RNA transcript, possibly due to an IRES‐mediated alternative translation mechanism.

To evaluate the impact of the reducing Δ40p53 expression on the fibrogenic process, LX‐2 cells were treated with the Δ40 gapmer (150 nM) for 24 h, followed by stimulation with TGFβ1 (5 ng/mL) for 24 and 48 h to monitor gene expression changes. The Δ40 gapmer successfully inhibited the mRNA expression of Δ40p53 by approximately 60%–85%, maintaining the suppressed expression for 48 h (Figure 3D). In addition, the fully spliced p53 transcripts (FLp53) without intron retention increased in cells treated with TGFβ1 compared to untreated cells, regardless of the presence of the Δ40 gapmer (Figure 3D). This suggests that the Δ40 gapmer inhibits Δ40p53 expression without affecting FLp53 expression. We then assessed the changes in mRNA levels of profibrogenic genes, including COL1A1, COL3A1, COL4A1, ACTA2, and TGFB1, in the TGFβ1‐treated LX‐2 cells (Figure 3E). Quantitative RT‐PCR demonstrated that COL3A1 and ACTA2 expression remained at similar levels to those in untreated control cells, while COL1A1, COL4A1, and TGFB1 significantly increased upon activation by TGFβ1 in both control and Δ40 gapmer‐treated cells. However, the overall expression levels of these genes were lower in cells where Δ40p53 expression was downregulated by the Δ40 gapmer compared to the control at both time points. Notably, western blot analysis confirmed that collagen type III was consistently downregulated by the Δ40 gapmer, and its increase by TGFβ1 activation was significantly constrained (Figure 3G).

Matrix metalloproteinases (MMPs) and tissue inhibitors of metalloproteinases (TIMPs) play essential roles in the pathogenesis of liver fibrosis by modulating ECM remodeling [41]. To investigate whether downregulation of Δ40p53 affects the expression of MMPs (specifically, MMP‐1, MMP‐2, and MMP‐9) and TIMPs (TIMP‐1 and TIMP‐2), we analyzed changes in these genes in LX‐2 cells transfected with either control or Δ40 gapmer, followed by vehicle or TGFβ1 treatment. Our qRT‐PCR data revealed that reduced Δ40p53 expression differentially affected MMP expression (Figure 3F), whereas TIMP‐1 and TIMP‐2 expression levels remained unchanged (data not shown). Targeting Δ40p53 resulted in approximately a two‐fold upregulation of MMP‐1 compared to the control. Following TGFβ1 activation, MMP‐1 significantly decreased relative to unstimulated conditions in both control and Δ40 gapmer‐treated cells. However, the level of MMP‐1 in Δ40 gapmer‐treated cells was notably higher than that observed in control cells. Furthermore, the downregulation of Δ40p53 significantly decreased MMP‐9 levels in both TGFβ1‐treated and untreated conditions, while Δ40p53 did not appear to impact MMP‐2 expression. These findings suggest that Δ40p53 selectively modulates MMPs involved in the fibrogenic process.

Western blot and qRT‐PCR analyses consistently demonstrated that the Δ40 gapmer suppressed α‐SMA expression, indicating that Δ40p53 may regulate LX‐2 activation in response to TGFβ1 (Figure 3E,G). Immunofluorescence staining further supported these molecular and biochemical findings, showing that TGFβ1 induces α‐SMA in control gapmer‐treated cells, while this induction is significantly reduced in Δ40 gapmer‐treated cells (Figure 3H). Collectively, these results suggest that Δ40p53 is a key regulator in TGFβ1‐induced gene expression, particularly in the modulation of profibrogenic ECM genes and proteins.

### 3.3 Blocking of Δ40p53 Translation Combined With Gapmer ASO Fully Inhibits Activation of LX‐2 Cells

**FIGURE 4:**
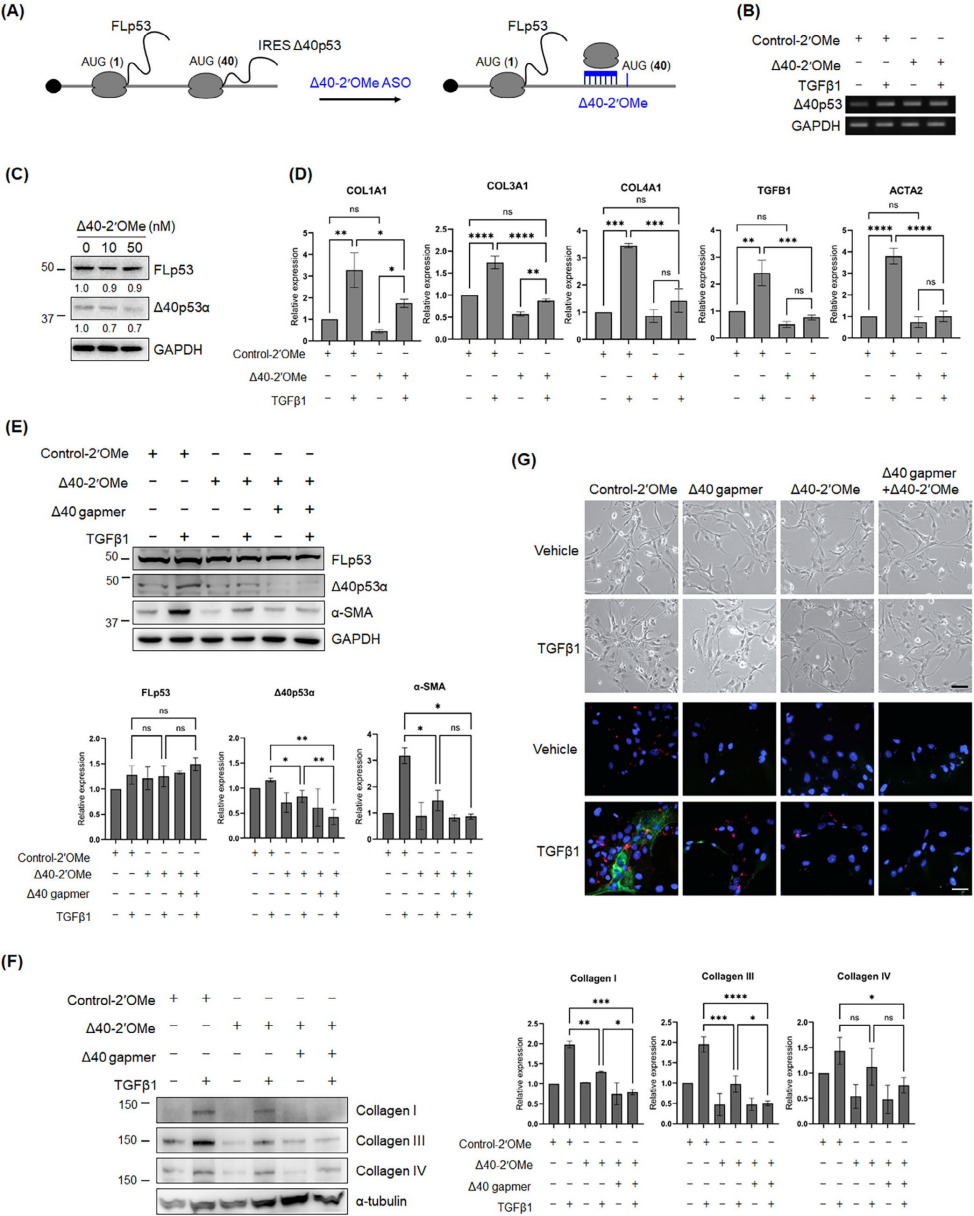
Combined treatment with Δ40 gapmer and 2′OMe enhances effects in TGFβ1‐stimulated LX‐2 cells. (A) Schematic showing the Δ40p53 isoform produced through internal ribosome entry site. The region blocked by 2′OMe ASO (Δ40‐2′OMe) is indicated. (B) Representative semi‐quantitative RT‐PCR showing Δ40p53 expression in LX‐2 cells treated with various concentrations of Δ40‐2′OMe. GAPDH was used as an internal control. (C) Western blot analysis of Δ40p53 protein levels in LX‐2 cells treated with the indicated concentrations of Δ40‐2′OMe. GAPDH served as a loading control. (D) qRT‐PCR analysis of profibrogenic ECM, ACTA2, and TGFB1 gene expression in control and Δ40‐2′OMe‐treated LX‐2 cells stimulated with either vehicle or 5 ng/mL TGFβ1 for 24 h (*N* = 3). GAPDH was used as an internal control. (E, F) Western blot analysis and quantification of profibrogenic ECM, FLp53, Δ40p53, and α‐SMA levels in LX‐2 cells treated with a combination of Δ40 gapmer and Δ40‐2′OMe, then stimulated with either vehicle or 5 ng/mL TGFβ1 for 24 h. Blots represent three independent experiments. GAPDH served as a loading control. The full‐blot images of FLp53 and Δ40p53 are shown in Figure S1B. (G) Representative phase images and immunofluorescence staining images of α‐SMA (green) and collagen III (red) in LX‐2 cells treated with a combined Δ40 gapmer and 2′OMe, then stimulated with either vehicle or 5 ng/mL TGFβ1 for 24 h. Nuclei are counterstained with DAPI. Scale bar, 25 μm. All quantitative data are presented as mean ± SD. *p < 0.05; **p < 0.001; ***p < 0.0001; ****p < 0.00001; ns, not significant [one‐way ANOVA with Dunnett's multiple comparison test (D–F)].

In addition to being generated from Δ40p53 transcripts that retain intron 2, the Δ40p53 protein can also be produced by an IRES‐mediated cap‐independent translational mechanism using an initiation codon in exon 4 (AUG 40) of the fully spliced p53 mRNA, without the intron retention [31, 32, 42] (Figures 1A and 4A). Studies have reported that IRES‐dependent translation of FLp53 and Δ40p53 isoforms is primarily prompted under stress conditions [29, 31, 39, 42]. We observed that the expression of profibrogenic genes remained elevated in Δ40 gapmer‐treated cells in response to TGFβ1, although their overall levels were lower than those in the control gapmer group (Figure 3E). Additionally, there was an increase in the fully spliced variant in the cells treated with TGFβ1, compared to untreated cells (Figure 3D). Based on these findings, we hypothesized that the IRES‐mediated alternative translation of Δ40p53 isoforms contributes to the inability of the Δ40 gapmer to fully downregulate the expression of profibrogenic genes upon TGFβ1 stimulation. To determine if the inhibition of cap‐independent translation of Δ40p53, combined with the Δ40 gapmer, could comprehensively downregulate fibrogenic gene expression, we used a steric blocking ASO (referred to as Δ40‐2′OMe herein) (Figure 4A), previously validated in published research to block the translation of Δ40p53 [39]. The steric‐blocking ASO contains a fully modified methyl base at the 2′ position of the sugar and prevents the interaction of mRNA with the ribosome for translation [43]. In Δ40‐2′OMe‐transfected LX‐2 cells, Δ40p53 RNA levels remained unchanged, while Δ40p53 protein levels decreased (Figure 4B,C). This confirms that the Δ40‐2′OMe ASO targets Δ40p53 translation without causing RNA degradation.

Quantitative RT‐PCR analysis demonstrated that LX‐2 cells treated with Δ40‐2′OMe exhibited significantly reduced RNA transcript levels of fibrotic markers (COL1A1, COL3A1, COL4A1, TGFB1, and ACTA2) compared to those treated with control ASO. Furthermore, Δ40‐2′OMe‐treated LX‐2 cells failed to increase fibrotic markers upon TGFβ1 activation (Figure 4D). Western blot analysis showed that the combined treatment of Δ40‐2′OMe with Δ40 gapmer significantly downregulated the expression of collagens and suppressed the activation of LX‐2 cells (Figure 4E,F). Notably, immunofluorescence staining of collagen type III and α‐SMA confirmed that the combined treatment of these two ASOs completely decreased the deposition of collagen type III and prevented the activation of LX‐2 cells (Figure 4G). These data suggest that the steric blocking of Δ40p53 with Δ40 gapmer significantly hampers the complementary mechanism of Δ40p53 expression and inhibits TGFβ1‐induced activation. Taken together, the upregulation of fibrogenic gene expression, namely α‐SMA, collagen I, III, and IV, was mitigated by using specific ASOs targeting Δ40p53, which suggests that Δ40p53 regulates fibrogenic gene expression in HSC cells.

### 3.4 An Increase of Δ40p53α Leads to an Upregulation of Profibrogenic Gene Expression

**FIGURE 5:**
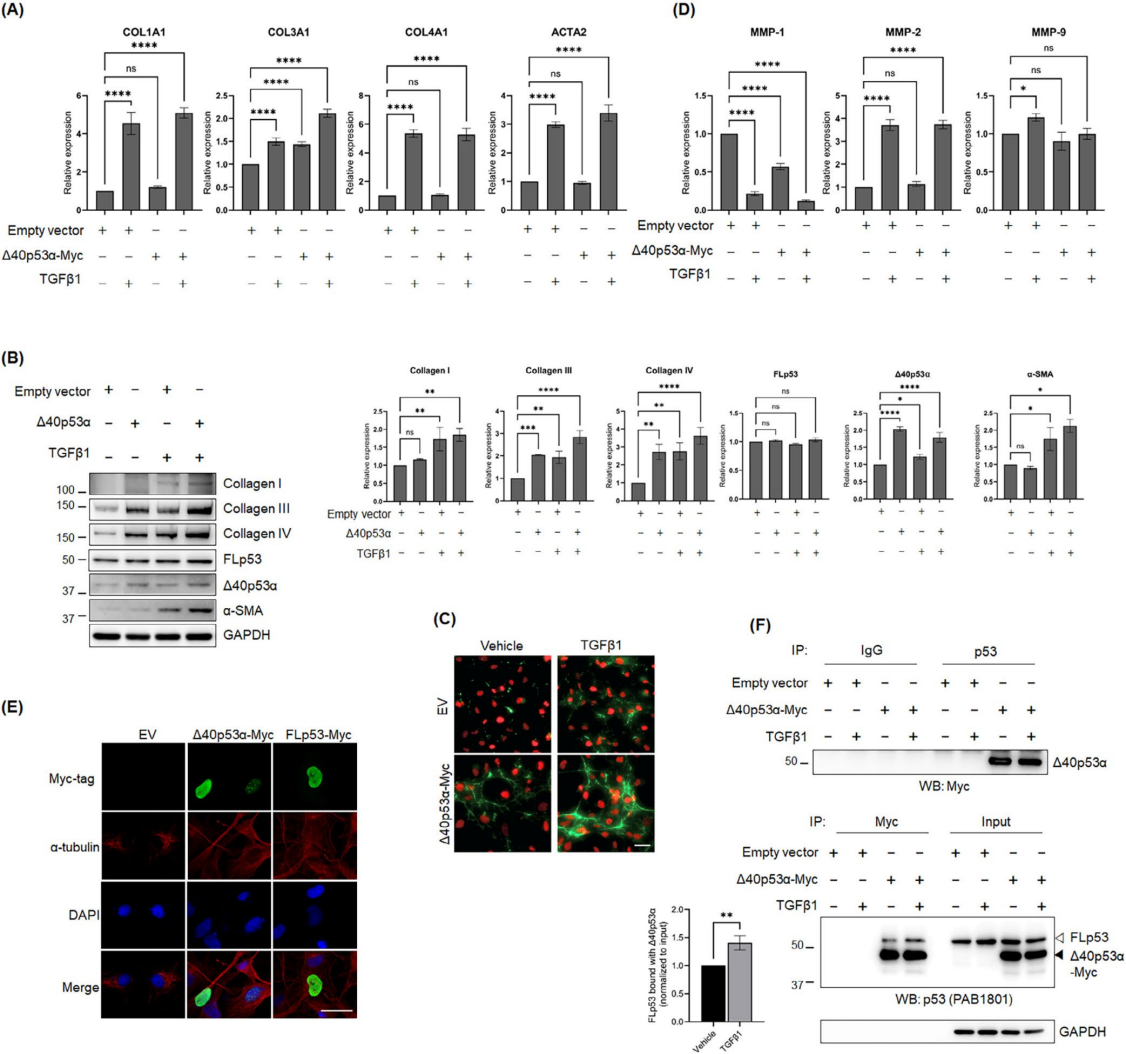
Elevated expression of 40p53 promotes upregulation of profibrogenic genes. (A) qRT‐PCR analysis of profibrogenic ECM and ACTA2 gene expression in LX‐2 cells overexpressing either empty vector or Δ40p53α, stimulated with vehicle or 1 ng/mL TGFβ1 for 24 h (*N* = 3). GAPDH was used as an internal control. (B) Western blot analysis and quantification of profibrogenic ECM, FLp53, Δ40p53, and α‐SMA levels in either empty vector or Δ40p53α‐overexpressing LX‐2 cells treated with vehicle or 1 ng/mL TGFβ1 for 24 h. Blots represent three independent experiments. GAPDH served as a loading control. The full‐blot images of FLp53 and Δ40p53 are shown in Figure S1C. (C) Representative fluorescence images of collagen III (green) and nuclei (red) in LX‐2 cells expressing either empty vector or Δ40p53α‐myc, treated with vehicle or 1 ng/mL TGFβ1 for 24 h. Scale bar, 25 μm. (D) qRT‐PCR analysis of MMP genes in LX‐2 cells overexpressing empty vector or Δ40p53α, treated with vehicle or 1 ng/mL TGFβ1 for 24 h (*N* = 3). GAPDH was used as an internal control. (E) Representative fluorescence images of cellular localization of Δ40p53α and FLp53. LX‐2 cells transiently transfected with myc‐tagged Δ40p53α or FLp53 were stained with anti‐myc tag antibody (green). α‐tubulin (red) marks the cytoplasm, and nuclei are counterstained with DAPI. Scale bar, 25 μm. (F) Co‐immunoprecipitation (co‐IP) analysis of FLp53 binding with Δ40p53α in LX‐2 cells transiently transfected with an empty vector or Δ40p53α‐myc, and treated with vehicle or 1 ng/mL TGFβ1 for 24 h. Cell lysates were incubated with antibodies against FLp53, Myc, or control IgG. Endogenous FLp53 binding to Δ40p53α was quantified by normalizing the FLp53 levels to Δ40p53α‐myc levels. GAPDH served as a loading control. All quantitative data are presented as mean ± SD. *p < 0.05; **p < 0.001; ***p < 0.0001; ****p < 0.00001; ns, not significant [one‐way ANOVA with Tukey's multiple comparison test (A, B, and D); two‐tailed unpaired *t*‐test (F)].

To investigate whether increasing Δ40p53α expression could counteract the observed inhibitory effect of ASOs on LX‐2 cell activation and profibrogenic ECM gene expression, we transiently transfected LX‐2 cells with Δ40p53α driven by the weak SV40 promoter to mimic the TGFβ1‐induced Δ40p53α levels (Figure 2A). Quantitative RT‐PCR revealed that Δ40p53α overexpression upregulated COL3A1 expression by 1.5‐ to 2‐fold, whereas COL1A1 and COL4A1 remained unaffected (Figure 5A). Consistent with the qRT‐PCR results, western blot analysis showed that the elevated Δ40p53α upregulated collagen III proteins by 2‐ to 3‐fold even without TGFβ1 stimulation, with further enhancement observed upon TGFβ1 stimulation (Figure 5B). Interestingly, western blot analysis also revealed increased collagen IV protein levels despite no significant changes at the transcript level (Figure 5B). Immunofluorescence staining confirmed the increased deposition of collagen III following Δ40p53α overexpression (Figure 5C). Notably, Δ40p53α overexpression exhibited higher α‐SMA levels in response to low‐dose TGFβ1 (1 ng/mL) compared to empty vector control cells (Figure 5A–C). This suggests that Δ40p53α may enhance TGFβ1 signaling in the activation of LX‐2 cells. Collectively, these results support that Δ40p53α plays a significant role in activating HSCs and regulating collagen III expression.

Given that reduced Δ40p53 expression differentially affected MMP expression (Figure 3F), specifically MMP‐1 and MMP‐9, we next assessed MMP expression in Δ40p53α overexpressing LX‐2 cells, both with and without TGFβ1 (Figure 5D). qRT‐PCR analysis showed that Δ40p53α overexpression resulted in a 2‐fold decrease in MMP‐1 compared to the control. Additionally, MMP‐9 was expressed at a level similar to that in empty vector controls when Δ40p53α was overexpressed, regardless of TGFβ1 activation. These results are aligned with the data we obtained from reduced Δ40p53 expression using Δ40p53 ASO (Figure 3F). Furthermore, Δ40p53α overexpression did not alter MMP‐2 expression compared to controls, confirming that Δ40p53α differentially regulates MMP expression. Collectively, our data suggest that elevated Δ40p53α expression promotes HSC activation and collagen III expression, and regulates ECM remodeling.

### 3.5 Δ40p53α Promotes Profibrogenic Gene Expression in Association With FLp53 and p‐Smad3 in Response to TGFβ1

Studies have shown that Δ40p53 isoforms have distinct roles and can modulate the activity of FLp53. Specifically, Δ40p53 isoforms can form complexes with FLp53, either antagonizing or cooperating with FLp53 function [29, 33, 35, 36]. We observed that Δ40p53α, like FLp53, is primarily localized in the nucleus, where it appears in both punctate spots and a diffuse pattern (Figure 5E). This nuclear localization suggests that Δ40p53α has distinct states that could reflect its interaction with nuclear components. To further investigate whether TGFβ1 stimulation influences the physical interaction between FLp53 and Δ40p53α, we performed co‐immunoprecipitation (co‐IP) in LX‐2 cells transiently transfected with either an empty vector or Δ40p53α‐Myc, followed by TGFβ1 stimulation. Co‐IP analysis revealed that Δ40p53α interacts with endogenous FLp53, and this interaction increased by approximately 1.5‐fold following TGFβ1 stimulation (Figure 5F).

**FIGURE 6:**
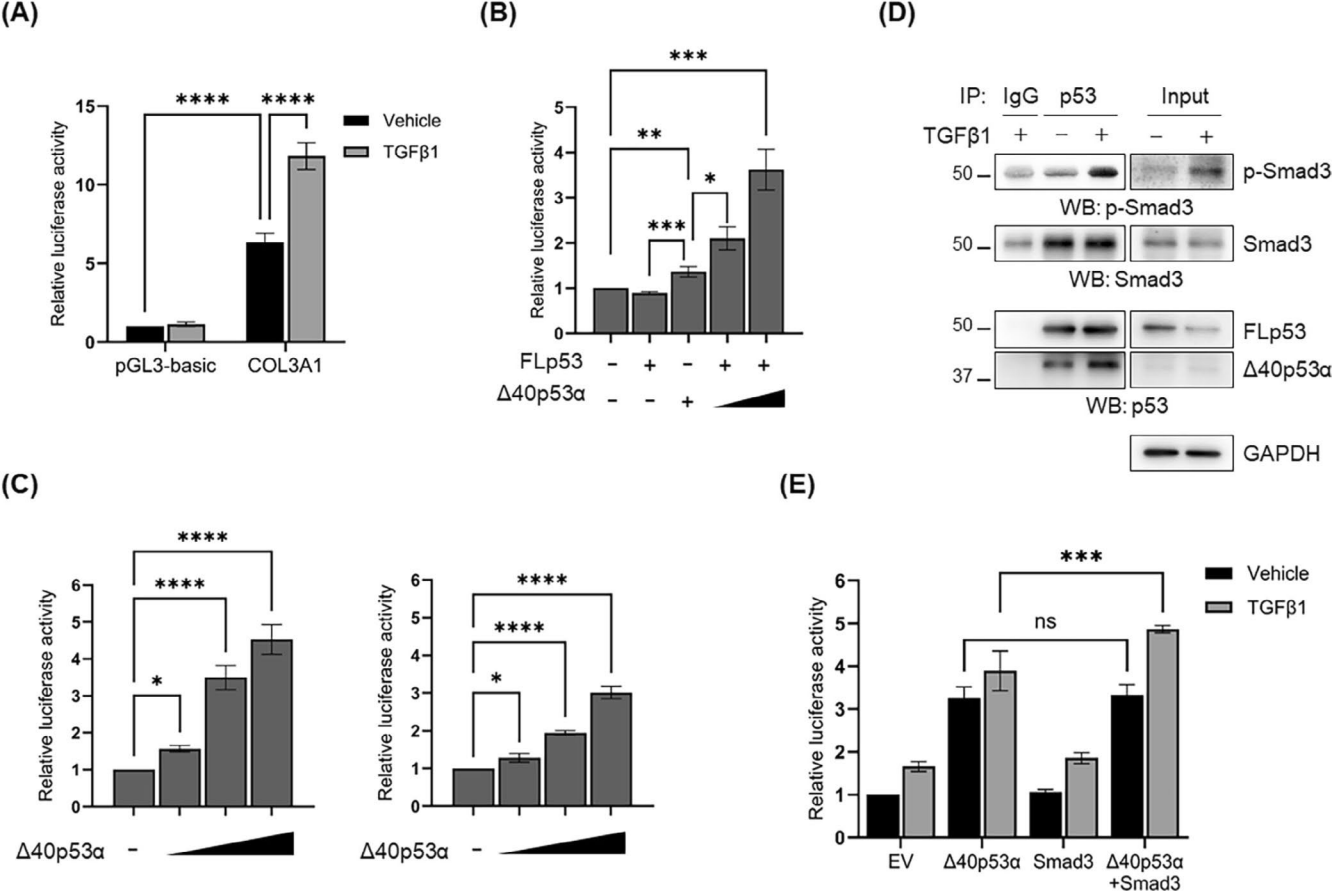
Δ40p53α promotes collagen III expression by interacting with full‐length p53 and p‐Smad3 in response to TGFβ1. (A) Luciferase reporter gene assay in transiently transfected LX‐2 cells with pGL3‐basic or pGL3‐COL3A1, followed by treatment with vehicle or 5 ng/mL TGFβ1 for 24 h. (B) COL3A1‐luciferase activity in transiently transfected HepG2 cells with pGL3‐COL3A1, FLp53 and Δ40p53α, either individually or together, with increasing amounts of Δ40p53α. (C) COL3A1‐luciferase activity in LX‐2 (left panel) and HepG2 (right panel) cells transiently expressing increased amounts of Δ40p53α. Firefly luciferase activity was normalized to *Renilla* luciferase activity. All data from three independent experiments were analyzed in triplicates. (D) Co‐IP analysis of p‐Smad3 binding to endogenous FLp53 and Δ40p53α in LX‐2 cells treated with vehicle or 5 ng/mL TGFβ1 for 24 h. Cell lysates were incubated with antibodies against FLp53 or control IgG, followed by western blotting for Smad3, p‐Smad3, FLp53, and Δ40p53α. GAPDH was used as a loading control. (E) COL3A1‐luciferase activity in transiently transfected LX‐2 cells with Δ40p53α and Smad3, either individually or together, followed by treatment with vehicle or 1 ng/mL TGFβ1 for 24 h. All quantitative data are presented as mean ± SD. *p < 0.05; **p < 0.001; ***p < 0.0001; ****p < 0.00001; ns, not significant [one‐way ANOVA with Tukey's multiple comparison test (B, C); two‐way ANOVA with a Tukey's multiple comparison (A, E)].

Given that collagen III is upregulated with the increased expression of Δ40p53α (Figure 5A–C) and that Δ40p53α localizes in the nucleus similarly to FLp53 (Figure 5E), we investigated whether Δ40p53α contributes to the transcription of COL3A1. A luciferase reporter assay using a COL3A1‐luciferase construct (−1100/+35 bp) showed that TGFβ1 induces approximately a 2‐fold increase in COL3A1 promoter activity (Figure 6A). Furthermore, analysis of COL3A1 promoter sequences with ALGGEN‐PROMO identified five high‐confidence p53 binding sites within the 1100 bp region upstream of the COL3A1 transcription start site (Tables [Link], [Link], and [Link], [Link]), suggesting a potential mechanism for direct interaction between p53 and the promoter. We then conducted overexpression experiments in LX‐2 cells to assess the transcriptional effects of Δ40p53α and FLp53 on COL3A1. Δ40p53α overexpression alone activated COL3A1 transcription, whereas FLp53 overexpression alone showed no significant effect. Notably, co‐expression of FLp53 with increasing amounts of Δ40p53α resulted in a dose‐dependent enhancement of COL3A1 transcription (Figure 6B). These results indicate that Δ40p53α directly activates collagen III transcription, and that this activation is enhanced by FLp53.

To evaluate whether this regulation was cell type‐specific, we expressed Δ40p53α in LX‐2 and HepG2 cells, which exhibit different basal levels of FLp53 (Figure 1D). Δ40p53α increased COL3A1 transcription in both cell types in a dose‐dependent manner, with higher transcription activity observed in LX‐2 cells compared to HepG2 cells (Figure 6C). We then examined whether Δ40p53α overexpression affects collagen protein expression in HepG2 cells. Western blot analysis revealed an upregulation of collagen III and IV protein levels in HepG2 cells overexpressing Δ40p53α, whereas collagen I levels remained unchanged (Figure S2). These findings are consistent with those observed in LX‐2 cells (Figure 5B), though the degree of upregulation was less significant in HepG2 cells. Notably, we found that HepG2 cells produce collagen I in response to TGFβ1. These results suggest that while HepG2 cells do not typically produce collagens, they may do so under fibrogenic conditions. Taken together, our findings indicate that Δ40p53α overexpression affects collagen protein production and that differences in basal FLp53 and Δ40p53α levels between HepG2 and LX‐2 cells correlate with variations in ECM gene expression.

Crosstalk between p53 and Smad‐dependent TGFβ signaling has been observed in various contexts [9, 10, 44]. In our study, co‐IP analysis demonstrated that the FLp53‐Δ40p53α complex associates with phosphorylated Smad3 upon TGFβ1 stimulation (Figure 6D). Furthermore, co‐expression of Δ40p53α and Smad3 upregulated COL3A1 promoter activity, and TGFβ1 further enhanced this effect (Figure 6E). These results indicate a potential interaction between the FLp53‐Δ40p53α complex and the Smad signaling pathway, contributing to the regulation of COL3A1 transcription.

**FIGURE 7:**
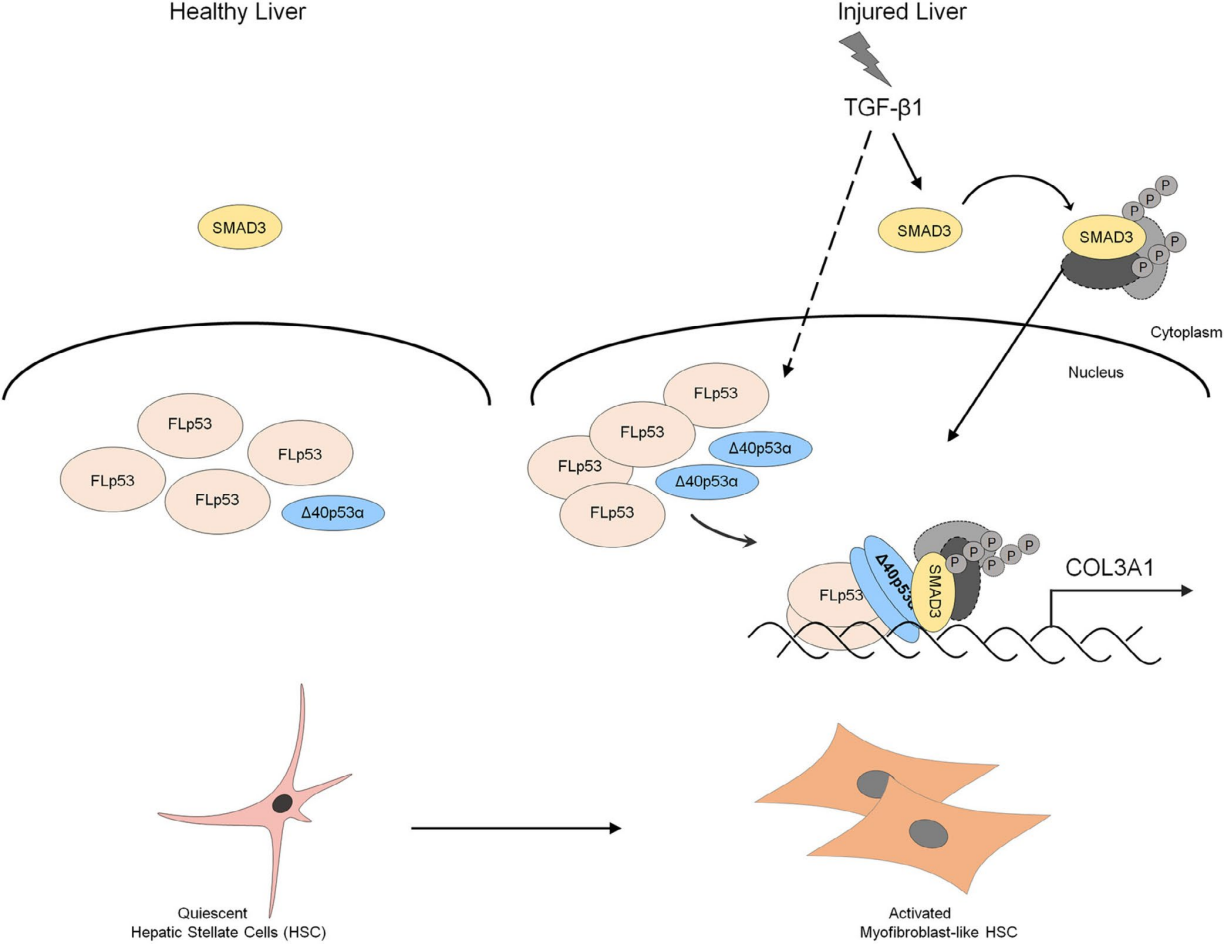
Proposed model for FLp53‐Δ40p53α‐Smad3 complex‐mediated COL3A1 expression in activated LX‐2 hepatic stellate cells. Following liver damage, TGFβ1 signaling upregulates Δ40p53α, promoting the formation of the FLp53‐Δ40p53α complex in hepatic stellate cells. In the nucleus, this complex associates with phosphorylated Smad3 (p‐Smad3), which is likely entering together with p‐Smad2 and Smad4 upon TGFβ1 activation [8]. The FLp53‐Δ40p53α‐p‐Smad3 complexes enhance COL3A1 transcription, leading to increased production and deposition of collagen III in activated hepatic stellate cells, ultimately, contributing to liver fibrosis.

Together, our findings support a model in which TGFβ1 stimulation increases Δ40p53α levels and its interaction with FLp53, promoting COL3A1 transcription in HSCs. This process is associated with an interaction between the FLp53‐Δ40p53α complex and p‐Smad3 (Figure 7). Our findings suggest that Δ40p53α may play a regulatory role in collagen III production and deposition in activated HSCs, a key feature of myofibroblast‐like HSCs.

## 4 Discussion

ECM plays a crucial role in the regulation of tissue‐specific functions and architecture [45]. In an injured liver, the tissue undergoes a repair process that balances wound repair and scar formation, which involves ECM remodeling. Disruption of ECM homeostasis is characterized by excessive deposition of ECM proteins such as collagen and fibronectin, which leads to pathological conditions such as liver fibrosis. The p53 tumor suppressor regulates cellular stress responses and is recognized as an important regulator of ECM gene expression. Although accumulating evidence has highlighted the role of p53 in profibrogenic ECM gene regulation, the specific contributions of its isoforms remain poorly understood. To address these gaps, we investigated the role of the p53 isoform Δ40p53α in modulating fibrotic gene expression. Our findings revealed that Δ40p53α isoform of p53 is a key regulator of ECM production during TGFβ1‐driven liver fibrogenesis. Δ40p53α enhances collagen III expression by forming complexes with FLp53 and phosphorylated Smad3, suggesting its pivotal role in promoting fibrogenic ECM accumulation.

Maintaining tight control over ECM homeostasis is essential for normal cellular behavior and overall physiological functions. Pathological changes in ECM production and deposition are major drivers of organ fibrosis [46]. Previous studies have demonstrated that p53 differentially regulates ECM gene expression, depending on the cellular context. For example, wild‐type p53 specifically represses fibronectin gene expression in HeLa cervical carcinoma and HepG2 hepatoblastoma cells [47]. Similarly, co‐expression of p53 with its isoform Δ133p53 suppresses fibronectin expression in mammary epithelial cells [23]. In mesenchymal cells, p53 inhibits TGFβ‐induced COL1A1 (collagen I) expression and other Smad‐dependent transcriptional responses [18]. In contrast to these suppressive effects, our results revealed a novel regulatory role of the p53 isoform Δ40p53α in promoting collagen III transcription. Specifically, Δ40p53α overexpression activated transcription, whereas FLp53 overexpression alone had no significant effect. Interestingly, the co‐expression of FLp53 and Δ40p53α synergistically enhanced COL3A1 transcription. This is particularly significant because increased levels of cross‐linked fibrillar collagens, such as collagen I and III, contribute to ECM stiffness, which can activate a positive‐feedback loop that further amplifies the profibrotic signaling pathway [48]. Our findings underscore a functional partnership between FLp53 and Δ40p53α in regulating collagen III transcription and strongly support their role in liver fibrosis.

MMPs, as critical regulators of ECM remodeling, play essential roles in maintaining tissue homeostasis and mediating fibrotic processes. Although previous studies have demonstrated that p53 regulates MMP gene expression, such as the downregulation of MMP‐1 (also known as interstitial collagenase or fibroblast collagenase) [19, 49], the role of specific p53 isoforms in this regulation remains largely unexplored. In our study, we found that Δ40p53 differentially modulates MMP expression, highlighting its potential influence on ECM remodeling during fibrogenesis. Specifically, knockdown of Δ40p53 resulted in upregulation of MMP‐1 and downregulation of MMP‐9, while MMP‐2 levels remained unchanged. Conversely, overexpression of Δ40p53α led to the opposite pattern, with downregulation of MMP‐1, upregulation of MMP‐9, and no significant effect on MMP‐2. These findings suggest that Δ40p53α exerts distinct regulatory effects on specific MMPs, supporting its role in ECM dynamics. Furthermore, we observed that the overexpression of Δ40p53α increased the protein levels of collagen I and IV, despite no significant changes in their mRNA levels. This discrepancy may be explained by the downregulation of MMP‐1, which could impair collagen degradation and potentially lead to the accumulation of collagen. Our findings suggest that Δ40p53 not only regulates ECM gene expression directly but also indirectly impacts ECM remodeling by modulating the balance between collagen synthesis and degradation. Overall, our results provide evidence that Δ40p53 plays a key role in ECM remodeling by differentially regulating MMP‐1 and MMP‐9. Future studies should investigate the role of Δ40p53 in the mechanistic pathways of MMP expression and activity to better understand its impact on ECM dynamics during the progression of fibrosis.

Although FLp53 relies on its N‐terminal transactivation domain (TAD1) for transcriptional regulation, previous studies suggest that Δ40p53 can also modulate gene expression despite lacking TAD1. Δ40p53 has been implicated in regulating genes involved in cell survival, apoptosis, and senescence [50, 51, 52], highlighting the ability of Δ40p53 isoforms to participate in gene regulation. Studies have shown that the intricate crosstalk between TGFβ signaling pathways and p53, particularly the interaction between p53 and Smad3 in various contexts [11, 18, 53]. Consistent with these findings, our study identified a novel regulatory mechanism of Δ40p53α in association with the FLp53‐p‐Smad3 complex in activated HSCs. This interaction suggests a previously unrecognized regulatory mechanism in TGFβ1‐mediated fibrotic gene expression. Consistent with previous reports [26], our co‐immunoprecipitation experiments confirm that Δ40p53 interacts with FLp53 to form a complex, which may function as a co‐activator that enhances the activity of p‐Smad3/2 complexes at the COL3A1 promoter upon TGFβ stimulation. This interaction likely stabilizes or enhances recruitment of transcription factor at ECM gene promoters, reinforcing the role of Δ40p53α in fibrotic gene regulation. Furthermore, we speculate that alternative transcriptional co‐factors may engage the TAD1 domain of FLp53 through protein–protein interactions with Δ40p53, thereby enhancing transcriptional activity. This mechanism could explain the observed upregulation of collagen III expression in activated HSCs, and places Δ40p53α as a key modulator of the liver fibrogenic process. Our findings raise the potential of targeting Δ40p53α to disrupt the FLp53‐Δ40p53α‐p‐Smad3 complex, thereby attenuating fibrogenesis without broadly inhibiting p53 or TGFβ signaling pathways.

An increasing number of studies have highlighted the critical role of p53 in organ fibrosis although its role varies with tissue type [54]. Evidence suggests that p53 contributes to organ fibrogenesis [10, 55, 56]. For instance, profibrogenic factors such as TGFβ1 and CTGF are upregulated in G2/M‐arrested tubular renal cells, and kidney fibrosis can be alleviated by a p53 inhibitor [57]. Similarly, elevated p53 levels have been observed in the fibrotic kidneys of mice, correlating with increased α‐SMA and CTGF. p53 promotes lung fibrogenesis by inducing apoptosis of alveolar epithelial cells [58]. In the liver, hepatocyte‐specific MDM2 knockout mice, which accumulate endogenous p53, develop fibrosis through the upregulation of CTGF [24]. Our findings align with these studies, further supporting p53's pivotal role in organ fibrosis through the regulation of profibrotic ECM gene expression. Specifically, we demonstrated that the FLp53‐Δ40p53α complex exerts a regulatory role in fibrogenesis by promoting collagen III production and deposition in activated HSCs.

However, there is contrasting evidence regarding the role of p53 in HSCs during liver fibrosis. For example, p53^−^/^−^ mice exhibit significantly more fibrotic tissue than WT mice [59], and HSC‐specific ablation of p53 reduces HSC senescence, leading to collagen accumulation and liver fibrosis [60]. The apparent contradiction in the role of p53 between these findings and our results may be due to the inherent heterogeneity of HSCs, as revealed by single‐cell RNA sequencing studies [61, 62, 63]. This heterogeneity could result in differential sensitivity and cellular responses to TGFβ1 among HSC subpopulations. Understanding this heterogeneity and elucidating the multifaceted role of p53 in this context are critical for advancing our knowledge of liver fibrogenesis.

The Δ40p53 isoform is known to be induced in response to various stress conditions, including DNA damage, cytotoxicity, serum starvation, and endoplasmic reticulum stress [29, 31, 42]. Our data indicate that Δ40p53 expression also increases in response to TGFβ1, a cytokine secreted by damaged tissues during inflammation. Given that TGFβ1 can trigger various stress responses depending on the tissue and cellular context [64], our observation of elevated Δ40p53 in cirrhotic liver tissues and activated LX‐2 human HSCs suggests that the increase in Δ40p53 is particularly relevant to stress conditions such as chronic liver injury. This conclusion is supported by the blocking of Δ40p53 translation with 2′‐OMe ASO, which reduced fibrogenic gene expression, and the combined treatment with Δ40 gapmer further decreased fibrotic markers and collagen III expression and deposition. Our data suggest that the IRES‐mediated, cap‐independent translation of Δ40p53 contributes to the upregulation of fibrogenic genes in response to TGFβ1. Overall, our findings imply that Δ40p53 plays a specific role in TGFβ1‐induced fibrosis and that targeting Δ40p53 with specific ASOs could potentially mitigate this fibrogenic effect.

Because gapmer ASOs can target both pre‐mRNA in the nucleus and mRNA in the cytoplasm, they may interfere with the regular splicing events of other p53 isoforms, which could potentially skew the results. Therefore, it is important to acknowledge that our data may carry a degree of bias owing to these ASO limitations, possibly affecting the interpretation of the biological effects attributed to Δ40p53 knockdown. However, the overexpression data for Δ40p53α clearly demonstrated that Δ40p53α has effects opposite to those observed with ASO treatment. This supports our conclusion that Δ40p53 promotes activation of LX‐2 cells and upregulation of profibrogenic gene expression, aligning ASO data with our proposed mechanism despite the limitations noted.

Post‐translational modifications within an epitope region can influence the antibody binding affinity by altering epitope accessibility [65]. In particular, the PAb1801 antibody recognizes amino acids 46–55 of p53 and is sensitive to phosphorylation at serine 46 and Pin1‐mediated proline isomerization at position 47, which is followed by phosphorylation at serine 46 [66, 67]. These modifications may induce conformational changes that either enhance or restrict antibody binding, thereby modulating PAb1801 immunoreactivity. Our data revealed slight variations in the detection intensity of FLp53 following TGFβ1 treatment, alongside the downregulation of the Δ40p53 isoform by ASOs, when probed with DO‐1 and PAb1801 antibodies (Figure S1). This deviation suggests a potential regulatory mechanism affecting antibody binding, possibly due to structural alterations or post‐translational modifications within p53 proteins. This brings attention to the need for further investigation to elucidate the molecular basis of this differential immunoreactivity of p53 and its isoforms, as well as their broader implications for function and regulation.

In summary, our findings revealed a functional partnership between FLp53 and Δ40p53α in regulating collagen III transcription, suggesting that Δ40p53α plays a critical role in the progression of liver fibrosis. By linking Δ40p53α to ECM remodeling and liver fibrosis, this study provides new insight into the mechanistic mechanisms driving fibrogenesis and highlights potential targets for therapies in fibrotic diseases.

## Author Contributions

Sun‐Young Lee and Yong‐Hwan Moon conceptualized and designed the research; Sun‐Young Lee and Yunseong Jang performed the research and acquired the data; Sun‐Young Lee, Hye‐Yeon Seok, and Yong‐Hwan Moon analyzed and interpreted the data. All authors were involved in drafting and revising the manuscript.

## Conflicts of Interest

The authors declare no conflicts of interest.

## Supporting information


Figure S1.

Figure S2.

Table S1.

Table S2.

Data S1.

## Data Availability

The data that support the findings of this study are available in Sections 2, 3, and/or Supporting Information of this article.
