# Supplementary figures and images for: A Novel Mechanism of the p53 Isoform Δ40p53α in Regulating Collagen III Expression in TGFβ1‐Induced LX‐2 Human Hepatic Stellate Cells
Source: FASEB J. 2025 Apr 15;39(8):e70541. doi: 10.1096/fj.202403146RR (PMC11999059; doi:10.1096/fj.202403146RR)

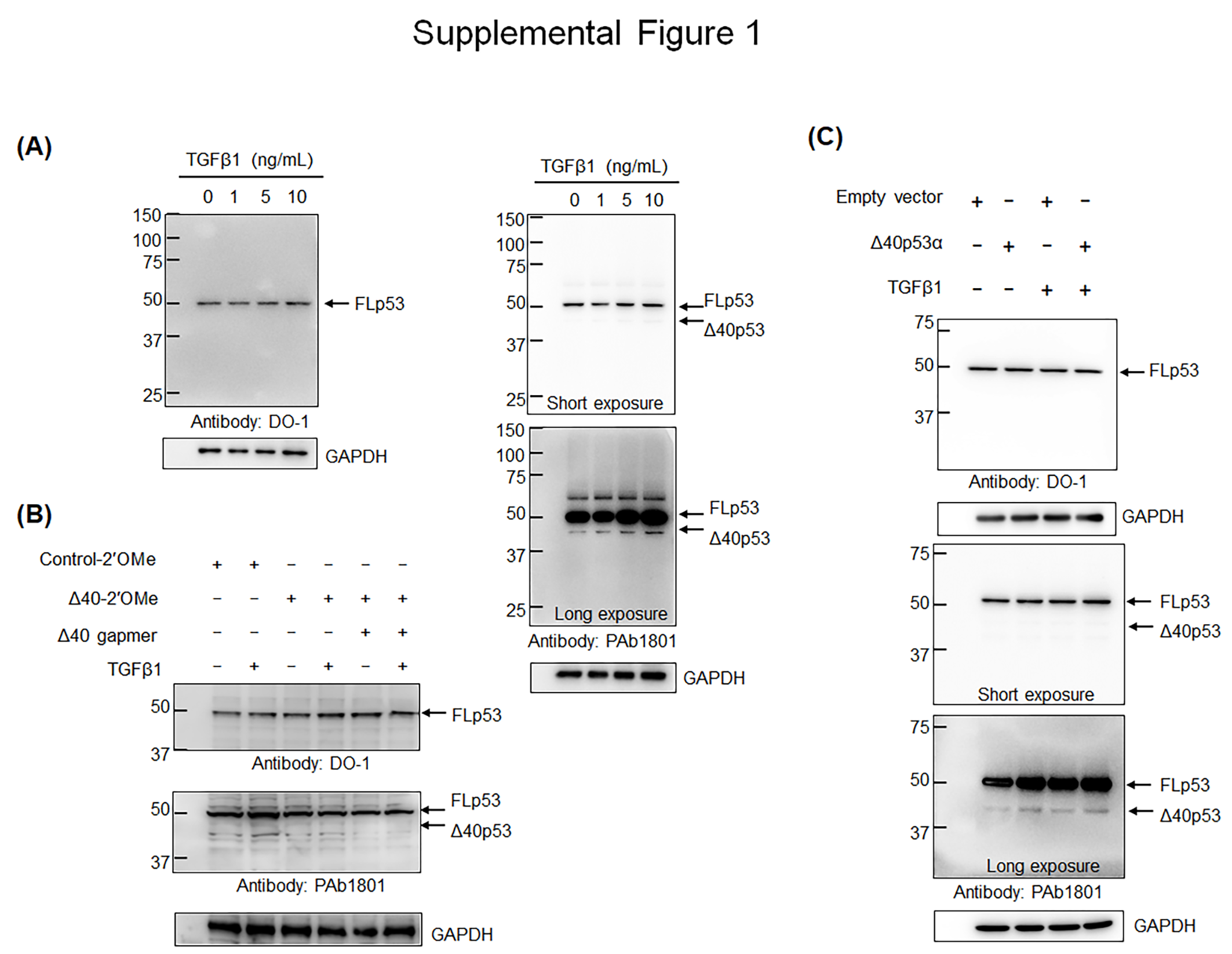

Supplement: Supplementary file 1 — Figure S1. [file FSB2-39-e70541-s002.tif]

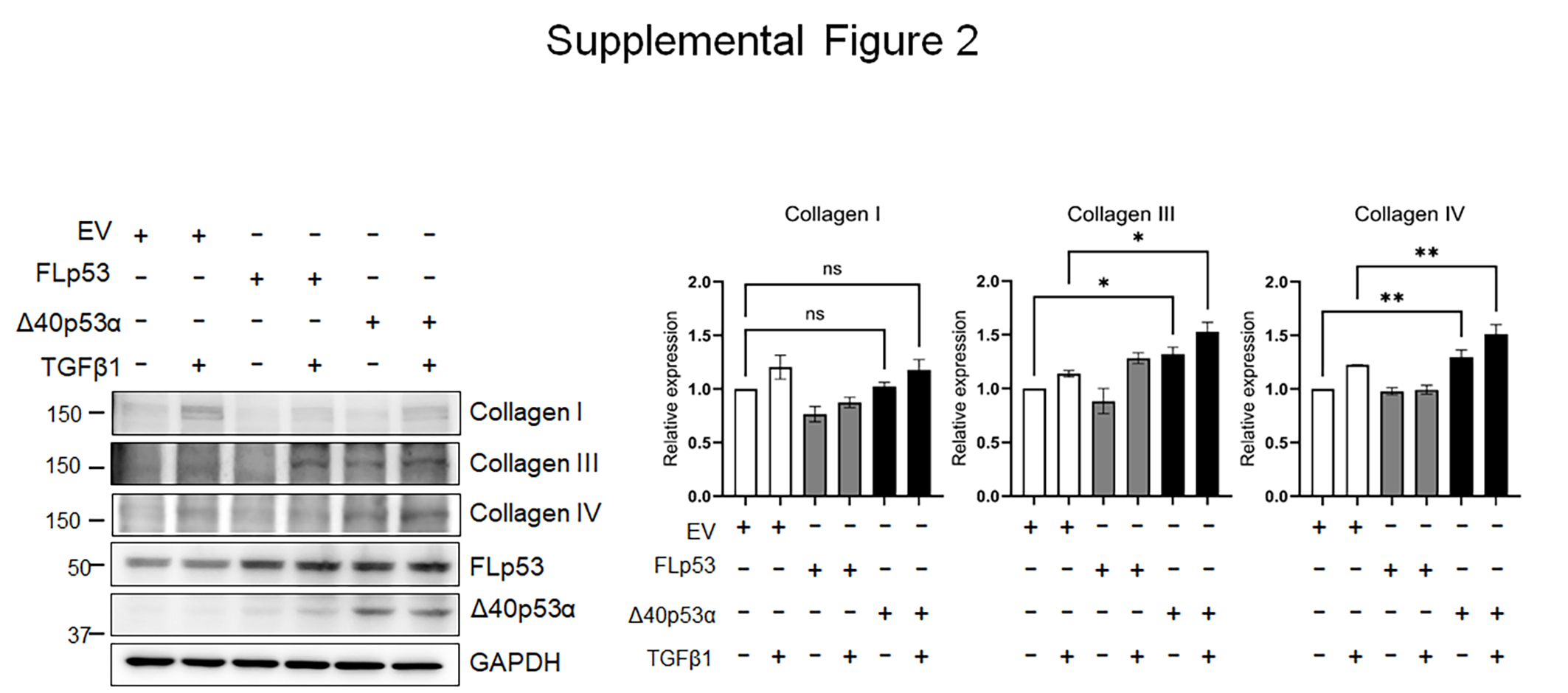

Supplement: Supplementary file 2 — Figure S2. [file FSB2-39-e70541-s004.tif]
