# Supplementary material for: A Novel Mechanism of the p53 Isoform Δ40p53α in Regulating Collagen III Expression in TGFβ1‐Induced LX‐2 Human Hepatic Stellate Cells
Source: FASEB J. 2025 Apr 15;39(8):e70541. doi: 10.1096/fj.202403146RR (PMC11999059; doi:10.1096/fj.202403146RR)
Supplement: Supplementary file 3 — Table S1. [file FSB2-39-e70541-s005.docx]

SUPPLEMENTAL TABLE 1 Summary of patient characteristics and diagnostic details

| Sample | M/F | Age | Sample Diagnosis | Pathological Verification  Notes from H&E review | Case  Diagnosis |
| --- | --- | --- | --- | --- | --- |
| N1 | M | 57 | Within normal  limits | 95% lobules, 5% portal triads | Colon adenocarcinoma |
| N2 | M | 73 | Within normal  limits | 95% lobules, 5% portal triads | Hepatocellular carcinoma |
| Cir1 | M | 50 | Cirrhosis of liver | 90% hepatocytes, 10%fibrous septa with triads; advanced stage 4 cirrhosis with chronic inflammatory infiltrate | Hepatocellular carcinoma |
| Cir2 | M | 56 | Cirrhosis of liver | 95% lobules, 5% portal triad; established cirrhosis | Metastatic melanoma |
| Cir3 | M | 43 | Cirrhosis of liver | 90% lobules, 10% triads; lesion; prominent portal fibrosis with nodule formation and marked mixed chronic inflammatory infiltrate, mild steatosis | Hepatocellular carcinoma |
