# Supplementary material for: A Novel Mechanism of the p53 Isoform Δ40p53α in Regulating Collagen III Expression in TGFβ1‐Induced LX‐2 Human Hepatic Stellate Cells
Source: FASEB J. 2025 Apr 15;39(8):e70541. doi: 10.1096/fj.202403146RR (PMC11999059; doi:10.1096/fj.202403146RR)
Supplement: Supplementary file 4 — Table S2. [file FSB2-39-e70541-s003.docx]

**SUPPLEMENTAL TABLE 2** COL3A1 promoter regions harboring p53 binding sites

| Factor | Start position | End position | String | Dissimilarity | RE equally | RE query |
| --- | --- | --- | --- | --- | --- | --- |
| P53 | -577 | -583 | TTTGCCC | 1.970013 | 0.27393 | 0.14019 |
|  | -492 | -498 | GGGCATA | 1.758307 | 0.27393 | 0.14019 |
|  | -355 | -361 | GGGCATC | 2.813291 | 0.36523 | 0.09870 |
|  | -57 | -63 | GGGCTGG | 3.750231 | 0.54785 | 0.11462 |
|  | 16 | 22 | CGGGCCC | 5.395490 | 0.45654 | 0.17220 |

RE: Random expectancy
