# Supplementary material for: A Novel Mechanism of the p53 Isoform Δ40p53α in Regulating Collagen III Expression in TGFβ1‐Induced LX‐2 Human Hepatic Stellate Cells
Source: FASEB J. 2025 Apr 15;39(8):e70541. doi: 10.1096/fj.202403146RR (PMC11999059; doi:10.1096/fj.202403146RR)
Supplement: Supplementary file 5 — Data S1. [file FSB2-39-e70541-s001.docx]

**Supplemental Figure 1. Full-blot images of FLp53 specifically recognized by the DO-1 antibody, as well as FLp53 and Δ40p53 analyzed using the PAB1801 antibody, are presented.** (A) The areas indicated by arrows on the blots were cropped and displayed in Figure 2C, showing the endogenously expressed FLp53 and Δ40p53α in LX-2 cells stimulated with various concentrations of TGFβ1. (B) The areas indicated by arrows on the blots were cropped and displayed in Figure 4E, showing the endogenously expressed FLp53 and Δ40p53α in LX-2 cells treated with a combination of Δ40 gapmer and Δ40-2′OMe, then stimulated with either vehicle or 5 ng/mL TGFβ1 for 24 h. (C) The areas indicated by arrows on the blots were cropped and displayed in Figure 5B, showing FLp53 and Δ40p53α in either empty vector or Δ40p53α-overexpressing LX-2 cells treated with vehicle or 1 ng/mL TGFβ1 for 24 h.

**Supplemental Figure 2. Overexpression of 40p53α increases the levels of collagen Ⅲ and Ⅳ in HepG2 cells.** Western blot analysis and quantification of collagen Ⅰ, Ⅲ, and Ⅳ in HepG2 cells overexpressing empty vector (EV), FLp53, or Δ40p53α, treated with vehicle or 1 ng/mL TGFβ1 for 24 h. Blots represent three independent experiments. GAPDH served as a loading control. All quantitative data are presented as mean ± SD. **P*<0.05; ***P*<0.001; ns, not significant [one-way ANOVA with Tukey’s multiple comparison test].
